# Enhanced gas separation factors of microporous polymer constrained in the channels of anodic alumina membranes

**DOI:** 10.1038/srep31183

**Published:** 2016-08-08

**Authors:** Ekaterina Chernova, Dmitrii Petukhov, Olga Boytsova, Alexander Alentiev, Peter Budd, Yuri Yampolskii, Andrei Eliseev

**Affiliations:** 1 Department of Materials Science, Lomonosov Moscow State University, Moscow, 119991, Russia; 2 Kurnakov Institute of General and Inorganic Chemistry, Russian Academy of Sciences, Moscow, 119991, Russia; 3 A. V. Topchiev Institute of Petrochemical Synthesis (TIPS) Russian Academy of Sciences, Moscow, 119991, Russia; 4 School of Chemistry, University of Manchester, Manchester, M13 9PL, UK

## Abstract

New composite membranes based on porous anodic alumina films and polymer of intrinsic microporosity (PIM-1) have been prepared using a spin-coating technique. According to scanning electron microscopy, partial penetration of polymer into the pores of alumina supports takes place giving rise to selective polymeric layers with fiber-like microstructure. Geometric confinement of rigid PIM-1 in the channels of anodic alumina causes reduction of small-scale mobility in polymeric chains. As a result, transport of permanent gases, such as CH_4_, becomes significantly hindered across composite membranes. Contrary, the transport of condensable gases (CO_2_, С_4_H_10_), did not significantly suffer from the confinement due to high solubility in the polymer matrix. This strategy enables enhancement of selectivity towards CO_2_ and C_4_H_10_ without significant loss of the membrane performance and seems to be prospective for drain and sweetening of natural gas.

Cost-effective and environmentally friendly gas separation is of vital importance in petroleum industry. Separation of condensable components (heavy hydrocarbons, carbon dioxide, water, hydrogen sulfide) from permanent gas streams (methane) is the common problem in natural and associated petroleum gas conditioning. Polymers with solubility-controlled selectivity allowing preferential transport of condensable components can be used for this purpose^1^. Polydimethylsiloxane and polymers with intrinsic microporosity (PIMs) are the common representatives of solubility-controlled polymeric materials. On the other hand the main disadvantage of these polymers is medium ideal and actual selectivity which needs to be improved. Possible change or even improvement of the membrane performance can be gained by the incorporation of selective layers into rigid porous supports. It is generally accepted that permeation of gases through a polymer material occurs by a molecule jumping between voids or free volume elements due to thermal movement of chain segments in rubbery polymers and small-scale mobility of functional groups in glassy polymers. It can be expected that by governing the motion of polymeric macromolecules it becomes possible to regulate permeability of gaseous components through the polymer membrane. One of the approaches to affect mobility of polymer chains is to use 2D geometric confinement, i.e. to put macromolecules into rigid and narrow cylindrical pores (Fig. 1a,b). Today, studying the influence of the geometric confinement on macromolecules dynamics, conformations and packing density has become a distinct field of research due to its importance in nanotechnology. Recently, the main focus was on the experimental determination of dynamics in polymer melts confined in rigid pores of anodic aluminum oxide (AAO)^2^,^3^. Polydimethylsiloxane (PDMS), polystyrene, and polyethylene oxide are the most frequently studied polymers in this field. It has been shown that the change of macromolecules dynamics depends not only on the confinement condition but also on the specific interactions between polymer chains and the pore walls. For instance, physical adsorption of PDMS macromolecules on the pore surface of anodic alumina gives rise to the formation of constrained layer consisted of a sublayer of adsorbed chains and an interphase of macromolecules with much slower dynamics than that of the bulk^3^. It can be assumed that introducing the polymer into rigid channels of inorganic support will cause changes in gas transport parameters. Manifestation of such phenomena can vary for penetrants with different solubility in the polymer. Permanent low-soluble gases would hardly permeate through constrained polymer chains (Fig. 1a). On the other hand, condensable gases with significantly higher solubility coefficients will cause swelling of the polymer enhancing small scale mobility. As a consequence, transport of condensable gases through spatially hindered macromolecules could be facilitated (Fig. 1b). Synergism of geometric confinement and solubility-based transport will promote selectivity of composite membranes towards condensable gases.

To the best of our knowledge there are no systematic and detailed studies elucidating the direct influence of 2D geometric confinement on the gas transport properties of polymers. In the present paper we test the applicability of geometric confinement for the improvement of membrane separation performance. Polymer of intrinsic microporosity (PIM-1) confined in the channels of anodic aluminum oxide was used as a model object. The main feature of the AAO microstructure is the system of hexagonally closely-packed cylindrical channels with low tortuosity and uniform pore-size distribution^2^,^3^,^4^. Anodic oxidation of aluminum in dissolving electrolytes enables to control the channel diameter, interpore distance and thickness of AAO films. AAO membranes exhibit high gas permeance for permanent (N_2_ permeance of 40 μm thick anodic alumina membrane is 50000 l bar^−1^ ∙ h^−1^ m^−2 ^5,6) and condensable (i-C_4_H_10_ permeance of anodic alumina membrane in capillary condensation regime exceed 500000 l bar^−1^ ∙ h^−1^ m^−2 ^7) gases. All these unique features enable AAO films to serve as excellent model systems for studying the geometric confinement influence on polymer transport characteristics. In the present work we were mainly interested in the development of composite membranes suitable for hydrocarbons separation, thus we consider high-free-volume microporous polymers for encapsulation to rigid AAO channels considered. We have decided in favor of PIM-1 because of its unique structural and transport characteristics. A special design of PIM-1 repeat unit based on spirobisindane gives rise to contorted macromolecules. Inefficient packing of PIM-1 chains results in high free volume elements fraction (22–24%) with the size less than 1 nm^8^,^9^. Gas solubility coefficients of PIM-1 are the greatest among all the polymers studied so far^10^. However, the studies of solubility controlled permeation of hydrocarbons in PIM-1 are discrepant^11^.

## Results and Discussion

**Figure 2:**
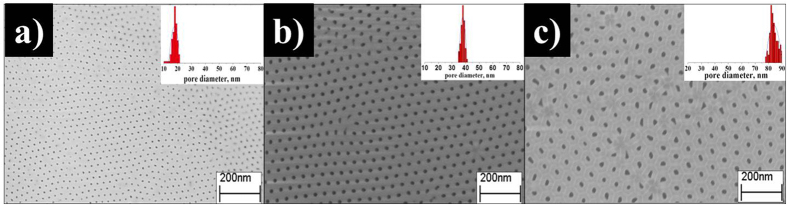
Microstructures of anodic alumina supports, prepared at different voltages: (**a**) 25 V (**b**) 40 V (**c**) 120 V.

To characterize the microstructure of AAO membranes, statistical analysis of electron microscopy images acquired from the bottom side of anodic alumina samples with different pore diameters was carried out (Fig. 2). Pore diameters were found to be narrowly distributed and nearly proportional to the oxidation voltage. The AAO membranes are known for linear dependence of permeability on the inverse square root of molecular weight of gases suggesting the dominance of the Knudsen diffusion mechanism^6^. Gas permeability of AAO membranes are represented in Table 1.

**Figure 3:**
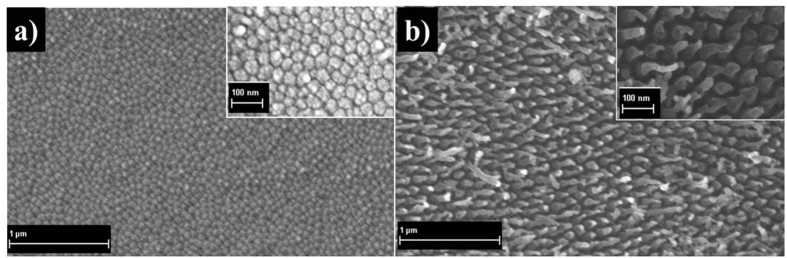
Polymer replicas of the composite membranes: (**a**) reference membrane C-40-F (**b**) composite membrane C-120. Insets: magnified areas of replicas.

To examine the microstructure of reference membrane and membranes partially impregnated with PIM-1, polymer replicas were detached from composite membranes by careful dissolution of AAO films in 0.5 M NaOH aqueous solution. Flat replicas of PIM-1 were observed in the case of the reference membrane revealing no infiltration of polymer into anodic alumina matrix (Fig. 3a). On the other hand, fiber-like nanostructures were found on the bottom side of selective layers of composite samples illustrating polymer infiltration into AAO channels (Fig. 3b). The same microstructure was obtained by imprinting of polyethylene terephthalate surface with anodic alumina stamp^12^. One should note that a detailed examination of PIM-1 replicas reveals the presence of nanochannels inside fiber-like nanostructures. A magnified area of polymer replica from sample C-120 (inset in Fig. 3b) exhibits the presence of polymeric nanotubes. To our opinion these hollow-fiber structures can notably affect gas transport properties of composite membranes but a detailed analysis of this point is beyond the main scope of the present study.

On the basis of SEM micrographs we can conclude that vacuum suction gives rise to partial polymer infiltration into rigid pores of AAO supports. As a result, the selective polymer layers of composite membranes consist of two sublayers (flat external sublayer and an internal sublayer with fiber-like nanostructures). Obviously gas transport parameters of flat external sublayer of composite membranes should comply those of bulk PIM-1, while permeability of internal sublayer can differ significantly introducing significant changes in the total membrane performance. It is convenient that comparison of polymeric membrane performances is performed in terms of permeability coefficients accounting the thickness of polymeric film. On the other hand, in the case of partially pore-filled membranes the exact determination of the total thickness of the selective films is rather unconventional problem. We failed to determine the precise length of the fiber-like nanostructures due to their partial hydrolysis of polymer in alkaline solution. Moreover, the length of polymer fibers depends on the penetration level of PIM-1 chloroform solution into the channels of AAO membranes and is different for certain channels diameters. Because SEM analysis allows only visualization of external sublayers, the total thickness of the polymer layer was also estimated by the luminescence spectroscopy (Table 2). Estimated thickness values obtained as maximum measured by PL and SEM were taken for calculation of composite membranes permeability coefficients. Porosity of AAO film was not taken in to account in estimations of polymer layer permeability as it provides major uncertainty in the resulting value. Due to uncertainty in determination of permeability coefficients we used permeance as well as permeability coefficients to examine composite membranes performance (Table 3).

The analysis of the obtained data shows significant decrease in permeance and permeability coefficients of composite membranes towards permanent gases as compared to reference membrane C-40-F. One should note the dramatic decrease of permeance, especially, for nitrogen and methane.

The permeability coefficients pass through a maximum for samples with pore supports of 35 and 49 nm and then decrease steadily. The calculated permeability coefficient of butane for C-25 sample is probably limited by the permeance of 100 μm AAO support (~8700 L ∙ m^−2^ ∙ bar^−1^ ∙ h^−1^).

**Figure 4:**
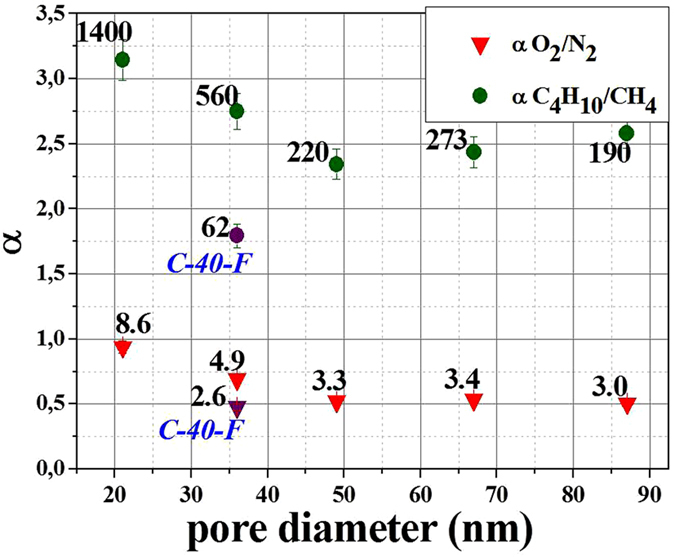
Dependence of pure-gas selectivity on pore diameter of AAO supports.

Substantial increase of pure-gas separation factors is reached for composite membranes as compared to reference membrane C-40-F (Fig. 4). The selectivity of composite membranes also significantly overpass the separation factor of bulk PIM-1 films (α C_4_H_10_/CH_4_ = 58)^11^. The separation factors of composite membranes with the smaller pore diameters are extraordinary high: the maximal ideal selectivity over 1400 for C_4_H_10_/CH_4_ gas pair, reached for C-25 composite membrane, exceeds substantially all the published selectivity values for polymers of intrinsic microporosity. Moreover, enhanced pure-gas O_2_/N_2_ selectivity of 8.7 was reached compared to maximum of 4.0 for freshly prepared PIM-1 membrane, activated in methanol^11^ and 5 for aged PIM-1^13^.

Pure-gas C_4_H_10_/CH_4_ and O_2_/N_2_ separation factors nearly monotonically decrease with increasing AAO pore diameters. This probably depends on both the polymer penetration depths and spatial constriction of macromolecular mobility. The second factor obviously dominates, which results in stronger selectivity enhancement for membranes with smaller pore diameters.

An explanation of significant rise in pure-gas selectivity can likely be attributed to the synergism of geometric confinement of PIM-1 macromolecules in AAO channels and high difference in solubility coefficients of permanent and condensable gases in PIM-1. The repeat unit of PIM-1 macromolecules contains two oxygen atoms in the dioxane ring and two CN-groups in the benzene ring enabling hydrogen bonding of polymer to the surface OH-groups of AAO pore walls. Adsorption of polymer macromolecules to the pore surface gives rise to slowing down of chains dynamics which results in the formation of an adhered surface layer. Adsorbed macromolecules are expected to affect the dynamics of polymer chains located closer to the pore center. The severity of the confinement increases with the reduction of the AAO pore diameters. This phenomenon is amplified by the intrinsic rigidity of PIM-1 macromolecules. At the same time, PIM-1 is characterized by extremely high solubility coefficients, in comparison to other polymers. The solubility coefficient of C_4_H_10_ in PIM-1 is about 90 cm^3^(STP) cm^−3^ bar^−1^, which corresponds to about 0.8 g of dissolved butane per 1 g of polymer at butane pressure of 1 bar^10^. At such a high concentration, molecules of the dissolved gas should affect mobility of polymer chains leading to polymer swelling. As a result, high permeability of dissolved gas across the swelled polymer should be observed. This explains high permeance of condensable gases in PIM-1. None of this holds for permanent gases. It can be assumed that the rate of butane mass transfer is less sensitive to the suppression of chains dynamics in confined PIM-1 compared to permanent gases and methane in particular. This gives rise to a substantial increase of pure-gas C_4_H_10_/CH_4_ and, to the less extent, CO_2_/CH_4_ separation factors.

A strong increase in selectivity of the composite membranes and sensitivity of the observed separation factors to the diameter of the pores implies that transport characteristics of confined PIM-1 differ from those of the bulk polymer. A possible role of such effects would be stronger if the pore radius is smaller than the radius of gyration R_g_ or end-to-end distance R_ee_ of individual polymer chains. Kuhn segments and the glass transition temperature can serve as indirect characteristics of these parameters. Despite we failed to find an assessed value of PIM-1 Kuhn segment the absence of glass transition temperature up to the point of thermal decomposition and ladder-like structure of PIM-1 macromolecules point to a high Kuhn segment value. Since unusual selectivity of PIM-1 embedded into AAO membrane is observed for narrower pores, it can be assumed that in the confined state the polymer chains could possess unusual conformations in the vicinity of pore walls. This interpretation is indirectly supported by the difference of the surface morphology of reference membrane with flat PIM-1 layer and composite membrane with fiber-like polymer nanostructures (Fig. 3). Such conformational change can be responsible for the observed transport properties of composite membranes.

Mixed gas CO_2_/CH_4_ measurements were performed to elucidate the influence of geometric confinement on polymer plasticization (Table 4). Reference membrane C-40-F and composite membrane C-40 aged for two months were used for the first measurements. It was revealed that the actual selectivity of the aged reference membrane is higher in comparison with the impregnated membrane. Selectivity of the composite membranes treated with methanol is quite different. The reference sample is prone to plasticization and its ideal selectivity is reduced. On the other hand, the ideal selectivity of the composite membrane increases giving evidence that the polymer in confined state is more resistant to plasticization under CO_2_. More detailed mixed gas measurements are in progress in our research group to get deeper insight into this problem.

**Figure 5:**
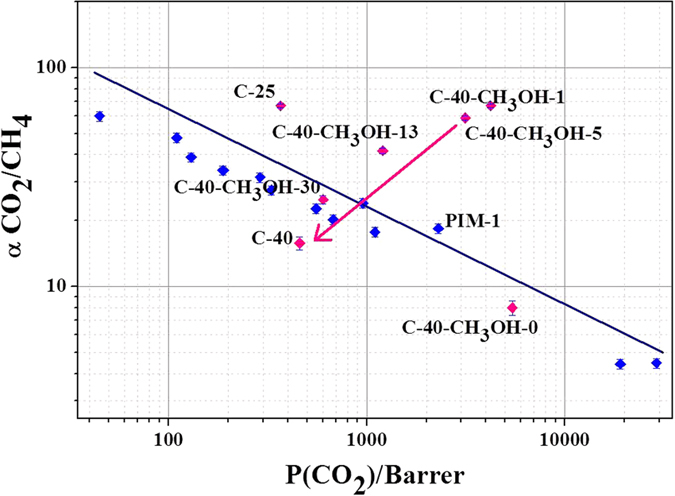
Selected composite membranes on the Robeson diagram.

It is well known that polymeric materials are prone to physical ageing. Especially this phenomenon is typical for thin films of highly permeable polymers. Diffusion of free volume elements to the surface of thin film occurs during physical ageing as an effect of polymer chains relaxation and packing^14^. One of the possible ways to slow down the process of physical ageing of polymeric films is annealing at temperature higher than the point of glass transition. However, this approach is not applicable for PIM-1 as this polymer exhibits no glass transition below decomposition temperature. An alternative way to rejuvenate PIM-1 after physical ageing is treatment with lower alcohols (especially CH_3_OH and C_2_H_5_OH). It has been shown that methanol treatment is the most effective way of revitalizing PIM-1^15^. Methanol molecules are able to form hydrogen bonds with polymer macromolecules leading to increase of chains dynamics which results in opening of free volume elements. In the present study we observed significant enhancement of as-prepared composite membranes permeance after methanol treatment (Table 5). One can note that the rise of permanent gas permeance leads to significant reduction of pure-gas separation factors. Further ageing of polymer in AAO matrix leads to colossal changes in the membrane performance with time due to rapid physical ageing of fiber-like selective layers. The points corresponding to the first days after methanol treatment go far over the upper bound on Robeson diagram (Fig. 5), while aged membrane indicates significant permeance decrease for both permanent and condensable gases. It can be assumed that geometric confinement promotes physical ageing. Obviously the process of chains packing is substantially enhanced in nanostructured polymer due to much smaller diffusion lengths as compared to bulk state. Packing of polymer chains also entails decrease of solubility coefficients of condensable gases.

It should be mentioned that the protocol of PIMs treatment is very significant for its gas separation properties. Fresh membranes casting should be followed by methanol treatment to withdraw residual solvent and to open free volume elements. On the other hand, methanol treatment switches polymeric chains to non-equilibrium state. Relaxation of macromolecules to stable conformations requires a certain period of time and depends on the film thickness. During long-term physical ageing polymer chains become more rigid and more close-packed and it can promote polymer solubility-selectivity. According to Pinnau^13^ highly aged PIM-1 and trypticene-PIM-1 have O_2_/N_2_ selectivity of 5 and 8.6 respectively and these numbers are in agreement with the O_2_/N_2_ permeance of C-25 (8.6) and C-40 membranes (4.9) suggesting the rigidification of PIM-1 in the confined state.

## Conclusions

The proposed strategy for composite membranes preparation enables substantial enhancement of the membrane pure-gas selectivity due to spatial constriction of polymer within the rigid porous framework. Chemical affinity of polymer macromolecules to pore walls of anodic alumina leads to slowing down of chains dynamics. High rigidity of PIM-1 macromolecules obviously resulting in large Kuhn segment, end-to-end distance, and persistence length favors confinement severity. As a result transport of permanent gases becomes hindered while condensable gases do not significantly suffer from geometric confinement due to high solubility in polymer matrix. This pathway opens wide opportunities for design of membrane transport parameters by governing segmental and small-scale mobility of polymer functional groups. It also can be realized by changing polymer chain conformations within a rigid matrix with pore diameters smaller than the size of the polymer Kuhn segment. We believe that further works on composite membranes containing polymers with large Kuhn segments will allow attaining substantial enhancement of polymer selectivity without significant loss of the membrane performance.

### Experimental Section

#### Preparation of composite membranes

The composite membranes based on porous AAO partially impregnated with PIM-1 were prepared by anodic oxidation of pure aluminum, followed by polymeric layer deposition using a spin-coating technique. High purity aluminum foil (99,999%, thickness 0.5 mm) was electrochemically polished in a solution containing H_3_PO_4_ (880 ml/l) and CrO_3_ (185 g/l) at 80 °C in pulsed mode (40 pulses, pulse duration is 3 s, current density is 0.65 A cm^−2^, voltage limit of 20 V). To prepare AAO films with different pore diameters an electrochemical oxidation at voltages ranged from 25 to 120 V was performed in a two-electrode cell using aluminum as an anode and Pt wire as a counter electrode. The electrolyte was continuously stirred during oxidation and its temperature was kept in the range of 0–4 °C. Continuous circulation of the electrolyte was arranged using a peristaltic pump (BT-300-2J, Longer Pump) in cases of anodization at 100 and 120 V. In order to obtain AAO supports with maximal permeability the oxidation process at 25 V and 40 V was performed in a two-step regime. A sacrificial layer of porous alumina formed after the first oxidation for 24 h was selectively removed in the solution of CrO_3_ (0.2 М) in H_3_PO_4_ (0.5 М) at 70 °С followed by second oxidation at the same conditions. The thickness of all membranes was coulometrically adjusted to 100 μm by controlling total electric quantity during the oxidation and assuming current efficiency of 90%. The preparation conditions are summarized in Table 1. To obtain AAO membranes gas-tightly fixed on aluminum foil, aluminum metal under porous oxide was selectively etched using CuCl_2_ (0.5 M) solution in HCl (5 vol. %). In order to open the pores at the bottom side of the membranes, the barrier layer was selectively dissolved in H_3_PO_4_ (25% wt.) under electrochemical control of pore opening. The prepared AAO membranes were partially filled with PIM-1 by vacuum clamping of AAO films during spin-coating of polymer solution using a KW 4A spin coater. Selective layers of PIM-1 were cast from chloroform solutions. Concentration of polymer solutions (0.25–2.0 wt. %) and rotation speeds (1200–5200 rpm) were optimized to from homogeneous defect-free films. As-prepared fresh composite membranes were examined for gas permeability. Rapid physical ageing in thin polymeric films was taken into account. It was observed that membranes permeability dramatically decreased during two weeks after preparation. Rejuvenation of the composite membranes permeability was achieved by swelling them in methanol for 30 min. For comparison, a composite membrane without polymer penetration into the channels of anodic alumina was prepared by spin-casting of PIM-1 solution onto AAO substrate without vacuum clamping (C-40-F sample).

#### Characterization of composite membranes microstructure

The microstructure of AAO and composite membranes was studied by SEM on Leo Supra 50 VP (21 kV, VPSE detector) and Carl Zeiss NVision 40 (5 kV, InLens detector) electron microscopes. The micrographs of AAO films were processed with ImageJ software in order to calculate pore diameters and pore-size distribution. To study the microstructures of composite membranes, AAO supports were carefully dissolved in 0.5 M NaOH and SEM analysis of the polymer replicas was performed. Due to experimental difficulties in accurate SEM determination of the PIM-1 layer thickness for composite membranes, luminescence spectroscopy was also applied to determine the total quantity of polymer deposited into (onto) AAO substrates. Luminescence of polymer was excited by a 473 nm 80 mW laser. Emission efficiency was measured by an Ocean Optics 65PRO spectrometer at maximal emission wavelength of 510 nm. PIM-1 film with thickness of 1.14 μm on optical glass support served as a reference sample. As thin (1–5 μm) PIM-1 layer absorption at λ = 473 nm does not exceed 50%, the film thickness can be estimated from PL emission intensity, assuming Beer-Lambert dependence of emitting centers quantity and neglecting concentration quenching. For permeability calculations the maximal value of thickness measured either by luminescence spectroscopy or SEM analysis was taken.

#### Gas permeability measurement

The permeability of AAO supports and composite membranes was tested for the gases in the following order: CH_4_, N_2_, O_2_, CO_2_, and n-C_4_H_10_. The data on permeance for all composite membranes were acquired next day (~24 h) to membranes preparation. Membrane samples were fixed in a two-compartment cross-flow cell. Gas fluxes at feed and permeate sides of the cell were controlled and measured by mass-flow controllers SLA5850 (Brooks, England). The pressure drop during gas transport was registered by PD-100-DI manometers (OVEN, Russia). Prior to measurements the whole system was evacuated to 10^−4^ mbar using T-Station 75 (BOC Edwards, England). Measurements were performed at ambient temperature (22 ± 2 °C) and atmospheric pressure of feed gas. In the upstream compartment of the cell continuous gas flow was organized during the whole time of measurement, thus providing constant boundary condition at the entrance of membrane surface. Typical experiment for any gas involved two stages: differential (open vacuum valve) with flow registered by mass flow controller and integral (closed vacuum valve) with flow measured from a pressure drop in the downstream cell. The duration of the first stage to achieve steady-state conditions typically did not exceed 1 hour. Then, the downstream compartment of the cell was closed with a vacuum valve and a pressure rise in the calibrated downstream cell was registered in time (typically in the pressure range of 0–0.2 bar), thus allowing measurement of the quantity of permeated gas. The permeances calculated both using differential and integral schemes were found to be identical for highly permeable gas (C_4_H_10_). Integral scheme was used to measure permeances of permanent gases to achieve better accuracy.

#### Mixed-gas permeation measurements

Mixed-gas permeation experiments were performed for binary CO_2_/CH_4_ mixtures. The carbon dioxide concentration in the feed stream was 10 vol. %. The upstream side of the membrane was in contact with the steady state flow of feed mixture at 1 bar, while the downstream side was swept by gas-carrier (helium). The retentate and permeate compositions were determined using Clarus 600 (Perkin Elmer) gas chromatograph with a thermal conductivity detector (TCD). The permeate/feed flow ratio was always kept below 1% so that the composition of the retentate was nearly the same as feed stream composition. The separation factor in the mixed gas experiments was determined using equation:


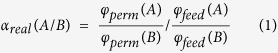


where *φ*_*perm*_*(A)* and *φ*_*perm*_*(B)* are mole fractions of the components A and B in the permeate and *φ*_*feed*_*(A)* and *φ*_*feed*_*(B)* are their corresponding mole fractions in the feed. Here the component A is carbon dioxide and component B is methane.

The mixed gas permeances were calculated by measuring concentrations of the penetrates in the gas-carrier and the total flow of this mixture. Partial pressure of penetrant on the upstream side of membrane was determined from the pressure and composition of the feed stream, and on the downstream side it was close to zero:


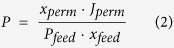


where *x*_*perm*_ and *x*_*feed*_ are the mole fractions of the gas components in the permeate and feed, respectively; *J*_*perm*_ is the total permeate flux and *p*_*perm*_ and *p*_*feed*_ are the absolute permeate and feed pressures.

## Additional Information

**How to cite this article**: Chernova, E. *et al*. Enhanced gas separation factors of microporous polymer constrained in the channels of anodic alumina membranes. *Sci. Rep*. **6**, 31183; doi: 10.1038/srep31183 (2016).

## Figures and Tables

**Figure 1:**
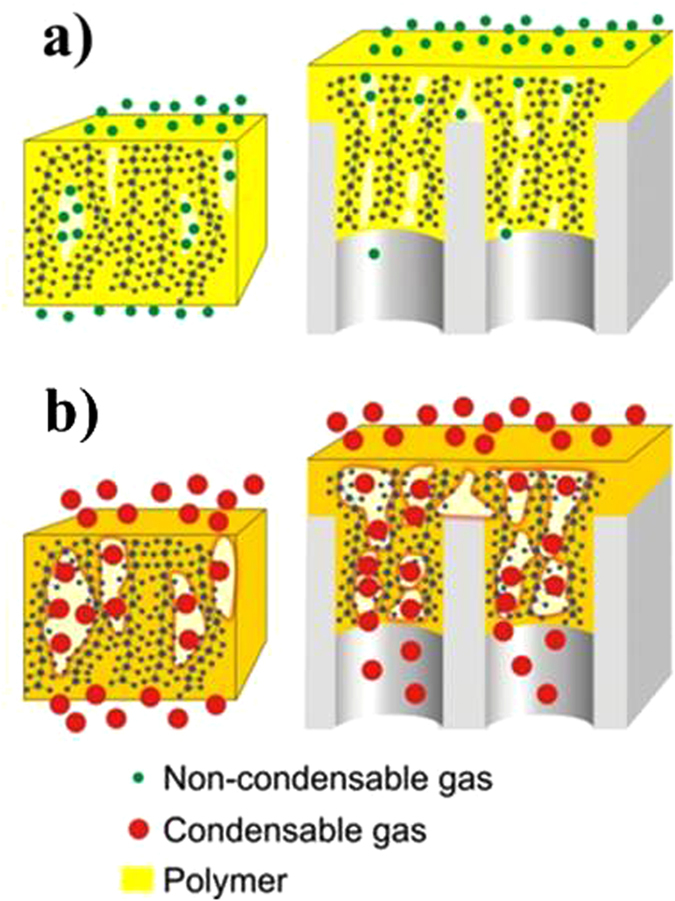
Schematic representation of: (**a**) transport of permanent gas molecules across free-standing (left) and confined (right) polymer; (**b**) solubility-controlled transport of condensable gas molecules across free-standing (left) and confined (right) polymer.

**Table 1:** General preparation conditions, microstructure parameters and permeance of AAO membranes.

Sample code	AAO-25	AAO-40	AAO-60	AAO-100	AAO-120
Electrolyte for anodization	0.3 M H_2_SO_4_	0.3 M H_2_C_2_O_4_			
Anodization voltage, V	25 (2 step)	40 (2 step)	60 (1 step)	100 (1 step)	120 (1 step)
Pore diameter, nm	21 ± 4	35 ± 4	49 ± 4	67 ± 10	82 ± 10
Average interpore distance, nm	55	104	145	216	265
Porosity, %	13.2	10.3	10.4	8.7	8.7
Gas	Permeance ∙ 10^3^, L ∙ m^−2^ bar^−1^ ∙ h^−1^				
CH_4_	14 ± 1	27 ± 3	32 ± 4	44 ± 3	48 ± 5
N_2_	13 ± 1	22 ± 3	25 ± 2	30 ± 2	38 ± 2
O_2_	11 ± 1	20 ± 2	23 ± 2	28 ± 3	33 ± 3
CO_2_	10 ± 1	18 ± 1	20 ± 1	25 ± 1	28 ± 2
C_4_H_10_	8.7 ± 0.5	17 ± 2	15 ± 1	24 ± 1	26 ± 1

**Table 2:** Effective thickness of the selective layers of composite membranes.

Sample	Emission intensity, a.u.	Thickness, μm	
		PL spectroscopy	SEM
C-25	1500	1.07 ± 0.2	2.40 ± 0.5
C-40	3350	2.39 ± 0.2	3.30 ± 0.7
C-40-1	3100	2.17 ± 0.2	3.00 ± 0.6
C-60	6000	4.28 ± 0.2	4.30 ± 0.9
C-100	2800	2.00 ± 0.2	0.93 ± 0.2
C-120	3500	2.49 ± 0.2	0.72 ± 0.1
C-40-F	9200	6.56 ± 0.2	5.87 ± 1.2

**Table 3:** Permeance and permeability coefficients of composite membranes.

Sample	Gas				
	Permeance, L m^−2^ h^−1^ bar^−1^ (permeability coefficients, Barrer)				
	O_2_	N_2_	CO_2_	CH_4_	C_4_H_10_
C-40-F	230 ± 11 (551 ± 27)	91 ± 4 (218 ± 11)	980 ± 49 (2346)	120 ± 6 (287 ± 4)	7460 ± 473 (17860 ± 400)
C-25	77 ± 37 (67 ± 3)	9 ± 4 (8 ± 4)	420 ± 18 (368 ± 18)	8 ± 2 (7 ± 3)	11400 ± 547 (9985 ± 479)
C-40	147 ± 7 (177 ± 9)	30 ± 2 (36 ± 2)	380 ± 19 (457 ± 23)	24 ± 1 (29 ± 1)	13500 ± 800 (16259 ± 830)
C-40-1	113 ± 5.7 (124 ± 6.2)	23 ± 2 (25 ± 1)	292 ± 15 (320 ± 16)	18 ± 0.9 (20 ± 1)	10000 ± 800 (10900 ± 547)
C-60	120 ± 6 (188 ± 9)	36 ± 2 (56 ± 3)	—	18 ± 2 (28 ± 2)	3970 ± 198 (6230 ± 311)
C-100	63 ± 3 (46 ± 2)	19 ± 1 (14 ± 2)	341 ± 17 (248 ± 12)	10 ± 1 (7 ± 1)	2760 ± 140 (2014 ± 102)
C-120	54 ± 3 (49 ± 2)	18 ± 1 (16 ± 3)	—	15 ± 1 (14 ± 1)	2820 ± 141 (2563 ± 128)

**Table 4:** CO_2_/CH_4_ mixed gas permeation properties of composite membranes, feed pressure 1 bar, T = 25 °C, mixture composition 10 vol.

Sample	Mixed gas selectivity (CO_2_/CH_4_)	Ideal selectivity (CO_2_/CH_4_)	Pure gas CO_2_ permeance, (l/m^2^ atm h)	Pure gas CH_4_ permeance, (l/m^2^ atm h)	Mixed gas permeance CO_2_, (l/m^2^ atm h)	Mixed gas permeance CH_4_, (l/m^2^ atm h)
C-40-F	7.9 ± 0.4	8.1 ± 0.5	980 ± 49	120 ± 6	950 ± 40	120 ± 5
C-40-1	20.8 ± 1.1	16.2 ± 0.7	292 ± 15	18 ± 0.9	274 ± 10	13.0 ± 0.5

%CO_2_/90 vol. %CH_4_.

**Table 5:** Permeance (L m^−2^ h^−1^ bar^−1^) and pure-gas selectivity of C-40 membrane.

Ageing duration, days	Sample	Gas			Selectivity	
		CO_2_	CH_4_	C_4_H_10_	CO_2_/CH_4_	C_4_H_10_/CH_4_
	C-40	380 ± 19	24 ± 1	13500 ± 800	15.8 ± 1.2	546 ± 35
0	C-40-CH_3_OH-0	4490 ± 90	560 ± 20	13970 ± 900	8 ± 0.5	149 ± 7
1	C-40-CH_3_OH-1	3500 ± 30	52 ± 10	7780 ± 200	67 ± 5	149 ± 10
5	C-40-CH_3_OH-5	2600 ± 50	44 ± 2	4900 ± 100	59 ± 4	111 ± 8
13	C-40-CH_3_OH-13	1000 ± 30	24 ± 1	2200 ± 100	41.6 ± 5.3	91.7 ± 7.2
30	C-40-CH_3_OH-30	500 ± 20	20 ± 1	750 ± 50	25 ± 3	37.5 ± 2.2
